# Comparative Study of the Quality of Life and Coping Strategies in Oncology Patients

**DOI:** 10.3390/ejihpe14020023

**Published:** 2024-02-06

**Authors:** Silmara Meneguin, Izadora Gama Alves, Heloiza Thais Felipe Camargo, Camila Fernandes Pollo, Amanda Vitoria Zorzi Segalla, Cesar de Oliveira

**Affiliations:** 1 Department of Nursing, Botucatu Medical School, São Paulo State University, São Paulo 18618-970, Brazil; s.meneguin@unesp.br (S.M.); isagamaalves13@gmail.com (I.G.A.); heloizathais@hotmail.com (H.T.F.C.); camilapollo@hotmail.com (C.F.P.); amanda.segalla@unesp.br (A.V.Z.S.); 2 Department of Epidemiology & Public Health, University College London, London WC1E 6BT, UK

**Keywords:** palliative care, cancer, coping strategies, quality of life

## Abstract

Background: Despite the current data on morbidity and mortality, a growing number of patients with a diagnosis of cancer survive due to an early diagnosis and advances in treatment modalities. This study aimed to compare the quality of life and coping strategies in three groups of patients with cancer and identify associated clinical and sociodemographic characteristics. Methods: A comparative study was conducted with outpatients at a public hospital in the state of São Paulo, Brazil. The 300 participants were assigned to three groups: patients in palliative care (Group A), patients in post-treatment follow-up with no evidence of disease (Group B), and patients undergoing treatment for cancer (Group C). Data collection involved the use of the McGill Quality of Life Questionnaire and the Ways of Coping Questionnaire. No generic quality-of-life assessment tool was utilized, as it would not be able to appropriately evaluate the impact of the disease on the specific group of patients receiving palliative care. Results: Coping strategies were underused. Participants in the palliative care group had poorer quality of life, particularly in the psychological well-being and physical symptom domains. Age, currently undergoing treatment, and level of education were significantly associated with coping scores. Age, gender, income, and the absence of pharmacological pain control were independently associated with quality-of-life scores. Moreover, a positive association was found between coping and quality of life. Conclusion: Cancer patients in palliative care generally report a lower quality of life. However, male patients, those who did not rely on pharmacological pain control, and those with higher coping scores reported a better perception of their quality of life. This perception tended to decrease with age and income level. Patients currently undergoing treatment for the disease were more likely to use coping strategies. Patients with higher education and quality-of-life scores also had better coping scores. However, the use of coping strategies decreased with age.

## 1. Introduction

### Background

Cancer is a main barrier to the growth in life expectancy throughout the world. According to the World Health Organization, cancer is a leading cause of death worldwide, accounting for nearly 10 million deaths in 2020, i.e., nearly one in six deaths [1]. Estimates from the International Agency for Research on Cancer showed that the world’s incidence was approximately 19.3 million new cases in 2020, with 10 million cancer deaths. Breast cancer in women has become the most commonly diagnosed cancer, with 2.3 million new cases (11.7%), followed by lung cancer (11.4%), colorectal cancer (10.0%), prostate (7.3%), and stomach (5.6%) [2]. The National Cancer Institute projected for the period from 2023 to 2025 the incidence of 704 thousand new cases in Brazil, with an emphasis on skin cancer (31.3%), prostate cancer (10.2%), and breast cancer (10.5%) [3].

Despite the current data on morbidity and mortality, more patients diagnosed with cancer are surviving due to early detection and advances in treatment options [4]. Surgery and radiotherapy are effective treatments for non-metastatic cancers, but they become less efficient when the cancer has spread throughout the body. For cancer that has spread, medications such as chemotherapy, hormone therapy, and biological therapies are preferred treatments, as they can reach all organs of the body via the bloodstream [5].

However, in some cases, a solo type of treatment may be used due to the varying levels of vulnerability of tumors to each therapeutic modality and the availability of treatments [6,7]. Despite the development of new and traditional therapeutic methods in cancer treatment, the side effects and toxicity of these treatments can negatively impact the quality of life of patients.

The World Health Organization (WHO) describes quality of life (QoL) as an individual’s perception of his/her position in life, concerning his/her personal goals, expectations, standards, and concerns, within the context of the culture and values of his/her society [8]. In terms of health, QoL refers to how individuals with chronic illness or any other condition perceive their health status and its impact on their daily activities [9,10]. In a clinical setting, QoL is related to the repercussions or treatment of diseases that can influence the perception of the construct [5].

Assessing QoL can help determine how a specific disease or intervention has impacted a patient’s life [11], making it a fundamental measure in oncology [12]. However, despite being a widely explored topic, the focus is often on controlling physical symptoms, with little attention given to the psychological, social, and spiritual aspects of care [4].

Furthermore, this measure becomes fundamental when a cure and the prolonging of life are not more possible [13]. Cancer is a chronic degenerative disease that poses considerable challenges for patients and their families, as the disease and its treatment constitute a threat to health and the integrity of the body. The diagnosis of malignant tumors is very often a stressful situation that brings up negative feelings and cognitive assessments influenced by previous experiences that result in the need for behavioral adjustment and coping with the disease [14,15].

There are various definitions of coping strategies in the literature. According to Lazarus et al. [16], coping is “the continuous cognitive adaptation and behavioural adjustment when dealing with specific internal and/or external demands that are perceived as exceeding an individual’s resources”. Coping is a dynamic process that involves a series of mutual responses between the individual and the environment, including intentional cognitive and behavioral actions aimed at mitigating the negative impact of a stressful event or situation [16].

The coping model establishes a link between internal and external factors, where challenges and threats (internal or external) and personal resources (e.g., a high perceived sense of self-efficacy and a socially supportive environment) are interrelated components [17].

The use of these strategies can contribute to coping with stressful situations or times of crisis that occur throughout life [18].

Coping strategies can be categorized into two main categories: problem-focused and emotion-focused. Problem-focused coping seeks to manage or modify the problem that is causing discomfort, dealing with the stressor in different ways, such as seeking information and planning actions. Emotion-focused strategies seek to regulate the emotional response to the problem [19].

Cancer is an emotionally challenging condition that can contribute to patients adopting negative coping strategies [20], as worry and sadness can be the main and continuous occurrence, after the diagnosis of this traumatic and stressful event [21,22].

A systematic review of symptoms in adults with lung cancer showed that 27% of these patients reported being in mental distress, and the majority described themselves as depressed, dysphoric, anxious, or with mood fluctuations [23]. Studies have shown that one-third of cancer patients experience anxiety and severe depression in their lives [24,25,26].

The diagnosis of advanced cancer leads the patient to experience existential suffering [27] and use multiple forms of coping strategies [28]. Studies supported the importance of questions about spirituality and existential concerns in coping with advanced cancer and its impact on quality of life [29,30].

Coping strategies can be decisive in QoL in cancer patients, as they affect both recovery and adaptation to possible disabilities resulting from it [31]. Studies have shown that the use of coping strategies is beneficial to patients with cancer in different stages of treatment, as such strategies contribute to a significant improvement in the quality of life [28,32,33].

Little has been explored in the literature about the association between coping and quality of life in oncology patients at various stages of their disease. Most studies address the topic focused on a type of cancer [34,35,36] or associated with resilience [37,38] and psychological suffering [39,40].

Therefore, this study aims to compare the quality of life and coping strategies among three cancer patient groups, identifying associated clinical and sociodemographic factors.

## 2. Materials and Methods

### 2.1. Participants

A comparative and cross-sectional study was conducted with three groups of cancer patients. This study was conducted at the outpatient service of the Oncology, Hematology, and Palliative Care Clinic of a public hospital in Brazil, between March 2018 and September 2019.

The eligibility criteria were male and female cancer patients aged 18 and older who agreed to participate in the study. Participants who were too emotional to continue the interview were excluded.

To test the study hypothesis, the participants were separated into three groups: Group A—patients in palliative care included consecutively after their first palliative medical appointment; Group B—patients who had completed cancer treatment six months to four years prior and had no signs of the disease; Group C—patients who were undergoing disease-modifying treatment for more than a month.

To determine the percentage of individuals who have MQoLQ and coping scores above average with a 10% margin of error and 95% confidence interval, we need a minimum sample size of 100 patients for each of the three groups. This was calculated as 1/M2, where M is the fixed margin of error.

### 2.2. Instrument

Three data collection instruments were applied. The first was a questionnaire collecting sociodemographic data. The second was the McGill Quality of Life Questionnaire (MQoLQ), which was translated and validated to the Portuguese language. It is a specific questionnaire for evaluating the quality of life in patients under palliative care. It has the largest number of validations in other languages, as well as the best measurement properties. This questionnaire is formed by 16 items distributed among five subscales: physical well-being, psychological well-being, existential well-being, support, and physical symptoms. There is an added item (Part 1) that measures overall quality of life (QoL) but is not considered in the calculation of the total MQoLQ score. The total score corresponds to the average of the five subscales, with scores closer to 0 denoting worse QoL and scores closer to 10 denoting better QoL [41] 20. No generic QoL assessment tool was used, as none would be capable of assessing the impact of the disease on the group of patients under palliative care. The alpha Cronbach of the instrument’s Brazilian version was 0.82.

The second instrument was the Brazilian version of the Ways of Coping Questionnaire created by Lazarus and Folkman, which is formed by 66 items distributed among eight domains: problem solving, escape/avoidance, self-control, positive reappraisal, confronting, withdrawing, social support, and accepting responsibility. The authors grouped these aspects into problem-focused coping (confronting and problem solving), emotion-focused coping (withdrawing, self-control, accepting responsibility, positive reappraisal, and escape/avoidance), and problem/emotion-focused coping (seeking social support). The items are scored on a Likert scale with four response options: 0 = “I did not use this strategy”; 1 = “I used this strategy a little”; 2 = “I used this strategy often”; and 3 = “I used this strategy a great deal”. There is no total sum score. The items are assessed according to the mean score of each factor [42]. In previous Brazilian studies, the alpha Cronbach of the instrument’s Brazilian version varied between 0.81 and 0.84 [43].

### 2.3. Procedure

To collect the data, a preliminary screening was conducted by one of the researchers on the medical records of patients who were scheduled for outpatient care. Each patient who met the criterion was invited to participate in the study. The interviews were conducted individually with each patient, after collecting his/her signed written consent, in a private setting, without the presence of an accompanying person, and before his/her medical appointment. In addition, the participants were assured of their anonymity and guaranteed that their data would be processed exclusively for scientific purposes. The interviews lasted approximately 40 min.

### 2.4. Data Analysis

First, all variables were analyzed descriptively. Proportions between groups were compared using the chi-squared test for nominal variables, the Kruskal–Wallis test for ordinal variables and ANOVA, and the Kruskal–Wallis test with Dunn’s post hoc test for quantitative variables. The comparison of median coping and quality-of-life scores between groups was performed using the Kruskal–Wallis test. Differences in coping and quality-of-life scores according to the clinical and demographic variables were evaluated using a generalized linear model. All analyses were performed with the aid of the IBM SPSS program, version 22, with the level of significance set at 5% (*p* < 0.05).

The internal consistency of the scale and its dimensions was evaluated using Cronbach’s alpha coefficients, with values higher than 0.70 considered acceptable [44].

This study was conducted using the checklist of “Strengthening the Reporting of Observational Studies in Epidemiology” (STROBE statement) [45] and received approval from the Human Research Ethics Committee of the Botucatu School of Medicine (certificate number: 2.598.647).

## 3. Results

Based on the eligibility criteria, 303 patients were selected. Three of them declined to participate (one in Group A and two in Group B) due to pain and the unavailability of time for the interview. Thus, the final sample was composed of 300 participants.

Women (n = 154; 51.3%), individuals living with a partner (n = 204; 68%), being catholic (n = 186; 62%), illiterate individuals and those with only primary school education (n = 183; 61%), and individuals with a household income equal to or less than three times the minimum monthly wage (n = 193; 64.7%) predominated in the sample. The participants in Group A were older (*p* < 0.01), had a lower level of schooling (*p* < 0.01), and resided in homes with a smaller number of residents. Gastrointestinal and mammary tumors predominated in Groups A and B, whereas hematological neoplasms predominated in Group C (Table 1).

Table 2 displays the descriptive statistics for the coping scores. Coping strategies were used little by the participants. There was a significant difference among groups for the problem-solving factor (*p* = 0.006), which was used more by the participants in Group C. In Group B, the escape/avoidance score was significantly lower (*p* < 0.001). No significant differences among groups were found for the mean scores of the other factors. Social support and withdrawal were the strategies most used by the participants of the study.

In Group A, the participants had a lower total quality-of-life score (median: 6.5) related to the other groups (*p* < 0.01), as well as lower scores in the psychological well-being (*p* < 0.01) and physical symptom (*p* < 0.01) domains.

Table 3 shows the results of the multivariate analyses using the generalized linear model for coping strategies and quality of life. Age (*p* < 0.001), belonging to Group C (*p* = 0.052), all levels of schooling (*p* = 0.029, *p* = 0.009, *p* = 0.049), and quality of life (*p* < 0.001) were significantly associated with the coping score. Age (*p* = 0.034), group (*p* = 0.015), sex (*p* = 0.042), income (*p* = 0.014; *p* = 0.005; *p* = 0.049), and coping (*p* < 0.001) were significantly associated with quality of life.

The Cronbach’s alpha of the McGill Quality of Life Questionnaire (MQoLQ) instrument was 0.85, and that of the Ways of Coping Questionnaire was 0.89; both are generally suitable for assessing accuracy [46].

Table 4 presents the factors that interfered with the participants’ perception of quality of life in the last two days. It is noted that family problems and pain stand out among the most influential in the perception of the construct.

## 4. Discussion

This study aimed to compare the quality of life and coping strategies among three cancer patient groups, identifying associated clinical and sociodemographic factors. Based on the current results, mean scores of the quality of life (QoL) were relatively good for the participants overall. However, those who were under palliative care had a poorer perception of the construct. This group showed substantial impacts in the physical well-being, psychological well-being, and symptom domains compared to the other groups.

In this context, the progression of the disease worsened the quality of life, highlighting the similarity between the construct and physical well-being perception. In fact, in a randomized study involving 733 patients with advanced or metastatic lung cancer undergoing different medicinal treatments, it was observed that the progression of the disease and symptoms contribute to a decreased quality of life (QoL) [47]. Data corroborated by another prospective study carried out with 105 cancer patients treated in an outpatient clinic of a tertiary hospital identified impairment of global well-being and low general quality of life of the participants [48].

When it comes to patients with advanced cancer, a particular approach to predicting their disease progression and commencing palliative care can enhance their quality of life. This is because the treatments suggested for such patients may not always alleviate their symptoms or stop the cancer from spreading, as supported by the cancer-related literature [49]. Hence, palliative care could be much more beneficial to them by avoiding and reducing their suffering and acknowledging their culture, spirituality, beliefs, and values.

In this study, the positive association between QoL and not receiving pharmacological pain control could be attributed to the fact that patients do not feel pain or that it is insignificant to the point of not interfering with their QoL. On the other hand, it is known that cancer pain management is still a challenge for health professionals, who encounter several obstacles related to the patient, such as a lack of knowledge about pain and its treatment, as well as incorrect conceptions about the use of opioids, often leading to underestimations of the pain and the proposed treatment [49].

The inverse association between income and QoL is in disagreement with findings described in a recent study involving cancer patients, which reported that individuals with low socioeconomic status, low level of schooling and advanced age had poorer health-related QoL [50]. Cancer can lead to financial difficulties for families as patients and caregivers often stop working after being diagnosed with the disease [51]. In this context, socioeconomic status, income, and level of education are factors associated with survival in patients with breast cancer, as documented in the literature [52].

The negative association found between QoL and age confirms the results of a previous study involving 608 older patients with cancer: age negatively impacted physical function and positively impacted emotional function [53]. The impact of age on QoL was also systematically studied in a review carried out with 6024 cancer patients which showed that QoL domains can be influenced by age [54]. Furthermore, it must be considered that comorbidities and physiological changes associated with ageing can influence the metabolism of anticancer drugs and their toxicity, thus impacting the quality of life [55].

Another important finding of the present investigation was a significant association positive between QoL and coping strategies; however, such strategies were little used by the study participants. This finding can be explained by theoretical frameworks that emphasize the role of these coping mechanisms in promoting positive affect, resilience, and overall well-being [53]. When examining the results for each subscale of coping, our study provides a nuanced understanding of the specific coping strategies that were used and can enhance patients’ well-being. These data are in line with a study carried out with 224 women with early-stage breast cancer in Iran wherein emotion-focused coping strategies were significant predictors of quality of life [34].

In the present study, social support was the most used coping strategy by the participants. Previous studies showed that women with breast cancer tend to use social support and withdrawal strategies more often than other coping mechanisms [54]. However, it is known that levels of social support, which decrease after cancer diagnosis and treatment, are crucial for cancer patients to return to their normal routine and recover their health-related quality of life. However, little is known about how this social support is provided, even though it has been linked to improvements in overall well-being and quality of life [55]. Furthermore, these authors emphasize that this support from the beginning of the disease and throughout follow-up would allow for specific interventions to improve recovery, especially in the most vulnerable patient groups.

Age is considered the most important predictive variable for coping strategies, as shown in the literature [56]. Our results showed a negative association between age and coping. This result can be attributed to the fact that older adults with cancer less frequently report psychological uncertainties, social stress, and situational stress compared to other age groups, as evidenced in recent research [57]. On the other hand, recent studies are showing that elderly cancer patients present hopelessness, resignation, safety-seeking behavior, and a reduced capacity to adapt to stress effectively [58]. Therefore, it is important to be more attentive to elderly patients upon arrival at oncology services to prevent the use of maladaptive strategies [59].

Participants in Group C, who were currently undergoing treatment, used coping strategies more frequently than other groups. This result is consistent with a previous study of 187 gastric cancer patients which found that emotion-focused and problem-focused coping strategies mediated the relationship between global and situational meaning and psychological well-being. For the authors, the relationship between changes in beliefs and goals, as well as psychological well-being, was also mediated by coping [60].

The positive association between coping and level of schooling can be attributed to the fact that a high level of education could improve the feeling of mastery and, thus, contribute to these patients having better access to information and understanding of medical information and the disease itself [59]. Furthermore, this difference in educational level reflects the disparity in economic status among individuals, thus subsequently affecting their health in various ways.

The theoretical foundations and empirical evidence discussed for each of our findings help to explain the mechanisms underlying the associations observed in this research. The discussion highlights the role of coping strategies for cancer patients and their influence on quality of life. Furthermore, it showed how these constructs are subjective and influenced by consistent sociodemographic predictors, as shown in the literature.

Although it was not the objective of this research to analyze the psychometric properties of the instruments, the reliability analysis carried out using Cronbach’s alpha showed that both instruments reached values above those estimated in the literature [44], which is 0.7. Concerning the Coping Strategies Inventory, in other Brazilian studies, Cronbach’s alpha values range from 0.81 to 0.89 [43,61]. In our study, the value found was 0.89, which can be attributed to the sample size, context in which it was applied, and patients’ profile.

Finally, the process of adjustment to cancer diagnosis and treatment appears to be dynamic and affected not only by the individual characteristics of each patient but also by sociodemographic and social factors. The expected family support during the disease does not always occur, and this trajectory is marked by negative feelings that impact quality of life.

### Limitations

This study has certain limitations that need to be taken into consideration. Firstly, the interviews were conducted at a single moment, which might not be enough to capture the full extent of changes experienced by individuals in different age groups. Secondly, the study was carried out in a single center, which had its unique characteristics. Moreover, the participants in the study had different types of cancer and clinical conditions, making them a heterogeneous group.

## 5. Conclusions

The study found that cancer patients receiving palliative care experience a lower quality of life compared to the other groups. Patients who do not use pain medication, patients with higher coping scores, and male patients reported a better perception of quality of life. However, this perception decreases in older patients and patients with higher income levels.

The average coping score was low, demonstrating that it was a strategy little used by the participants. Among the strategies used, social support prevailed. In our sample, patients who undergo treatment for cancer currently and have higher education levels and quality-of-life scores had better coping. However, the use of this strategy decreases in older patients.

Although this research provides valuable insights into the practical relevance of the results in oncology, it is considered important to highlight that this is an observational study. Therefore, we cannot establish a causal relationship concerning specific responses and the disease. On the other hand, the participants’ reports gave visibility to family problems, suffering, and limitations imposed by the disease, thus influencing the perception of QoL. The key findings from this study serve as a foundation for further exploration of the relationships between coping strategies, spiritual coping, symptoms of depression, anxiety, and stress among patients with cancer. It also offers support to health professionals to direct care strategies aimed at the real needs of these cancer patients that often go unnoticed or are little known.

## Figures and Tables

**Table 1:** Sociodemographic and clinical characteristics of participants. Botucatu, SP, Brazil, 2020.

Variable	Group			
	A	B	C	
Age (years)				
Median	62.0	56.0	56.0	
(p25–p75)	(52.2–69.0)	(41.2–67.0)	(45.0–64.0)	<0.011 ^1^
Time since diagnosis				
Median (months)	23.0	16.5	4	
(p25–p75)	11–42.3	9–28.3	3–10	*p* < 0.01 ^3^
Sex				
Female	50 (50.0)	54 (54.0)	50 (50.0)	
Male	50 (50.0)	46 (46.0)	50 (50.0)	0.81 ^2^
Marital status				
Without partner	36 (36.0)	31 (31.0)	29 (29.0)	
With partner	64 (64.0)	69 (69.0)	71 (71.0)	0.58 ^2^
Religion				
Catholic	66 (66.0)	59 (59.0)	61 (61.0)	
Non-Catholic	34 (34.0)	41 (41.0)	39 (39.0)	0.56 ^2^
Schooling				
Illiterate/primary school	78 (78)	50 (50.0)	55 (55.0)	
High school	18 (18)	34 (34.0)	28 (28.0)	<0.01 ^3^
University	4 (4)	16 (16.0)	17 (17.0)	
Household income *				
Less than 1 to 3× monthly minimum wage	76 (76.0)	59 (59.0)	59 (59.0)	0.05 ^4^
4 to 10× monthly minimum wage	20 (20.0)	34 (34.0)	40 (40.0)	
>10× monthly minimum wage	4 (4.0)	7 (7.0)	1 (1.0)	
Primary diagnosis				
Hematological	10 (10.0)	23 (23.0)	27 (27.0)	
Gastrointestinal	19 (19.0)	26 (26.0)	21 (21.0)	
Breast	16 (16.0)	24 (24.0)	33 (33.0)	<0.001 ^2^
Prostate	12 (12.0)	2 (2.0)	3 (3.0)	
Genitourinary	13 (13.0)	7 (7.0)	14 (14.0)	
Respiratory	15 (15.0)	5 (5.0)	1 (1.0)	
Other	15 (15.0)	13 (13.0)	1 (1.0)	

^1^ ANOVA + post hoc Tukey; ^2^ chi-squared test; ^3^ Kruskal–Wallis + post hoc Dunn; ^4^ Kruskal–Wallis; * monthly minimum wage = BRL 1100 (USD 196).

**Table 2:** Distribution of median (25th–75th percentile) and mean (SD) values of coping factors and quality-of-life domains among groups studied. Brazil, 2020. Coping.

	Group			
	A	B	C	*p*
Factors	Median (p25–75)	Median (p25–75)	Median (p25–75)	
Problem solving	1.4 (0.8–1.7)	1.6 (1.0–2.0)	1.7 (1.2–2.0)	0.006
Escape/avoidance	1.4 (1.1–1.7)	1 (0.6–1.4)	1.2 (1.0–1.5)	0.001
Positive reappraisal	1.7 (1.1–2.1)	2 (1.4–2.2)	1.9 (1.4–2.1)	0.030
Confronting	0.8 (0.7–1.0)	1 (0.7–1.2)	0.8 (0.7–1.0)	0.227
Withdrawing	1.9 (1.3–2.3)	2 (1.5–2.3)	2 (1.5–2.3)	0.502
Self-control	1.3 (1–1.7)	1.3 (1.0–1.9)	1.4 (1.0–1.9)	0.156
Seeking social support	2 (1.7–2.3)	2.2 (1.8–2.5)	2.2 (1.8–2.5)	0.351
Accepting responsibility	0.7 (0.5–1.2)	0.7 (0.2–1.2)	0.7 (0.5–1.2)	0.992
Quality of life				
Physical well-being	7.0 (4.0–8.0)	8.0 (7.0–9.0)	8.0 (7.0–9.0)	<0.012
Psychological well-being	3.8 (1.3–7.6)	7.0 (5.3–8.5)	7.0 (3.9–8.8)	<0.012
Existential well-being	8.3 (7.0–9.0)	8.8 (8.2–9.2)	8.8 (8.3–9.3)	<0.012
Support	9.0 (7.0–10.0)	8.5 (7.0–10.0)	9.0 (8.0–10.0)	0.372
Physical symptoms	5.0 (3.6–6.7)	8.5 (6.7–10.0)	7.7 (6.7–9.5)	<0.012
Score Total	6.5 (4.9–7.8)	7.9 (7.2–8.6)	8.0 (7.3–8.7)	<0.012

**Table 3:** Generalized linear model for coping and quality-of-life scores. Botucatu, SP, Brazil, 2021.

Variables	Coping		Quality of Life	
	β Coefficient	*p*	β Coefficient	*p*
Age	−0.2488	<0.001	−0.0148	0.034
Months since diagnosis	0.0204	0.500	2.75 × 10^−4^	0.922
Group				
A vs. B	1.2430	0.731	−0.8226	0.015
C vs. B	4.6201	0.052	0.1242	0.572
Sex (ref. male)	−0.8576	0.655	0.3643	0.042
Marital status				
(ref. without partner)	−0.3488	0.866	0.1984	0.300
Schooling (ref. illiterate)	16.0607	0.029	0.329	0.063
Income (Ref. > 10× monthly min wage)	−5.0835	0.482	−1.6488	0.014
Pain control				
None vs. pharmacological	−0.2901	0.933	0.6618	0.039
Quality of life	0.939	<0.001		
Coping			0.942	<0.001

**Table 4:** Answers referring to part D of the McGill Quality of Life Questionnaire. Factors that interfered with the perception of quality of life in the last two days. Botucatu, SP, Brazil, 2021.

Factors	N (%)
Family problems	37 (12, 3)
Pain	25 (8, 3)
Loneliness	21 (7, 0)
Discouragement/sadness/nervousness	20 (6, 6)
Being sick	18 (6, 0)
Fear of disease recurrence	14 (4, 6)
Dependence on others for daily activities	12 (4, 0)
Treatment sequelae (scar, loss of visual acuity, change in post-treatment habits, ostomy)	12 (4, 0)
Use of hospital medical devices (catheters)	11 (3, 6)
Problems with eating	10 (3, 3)
Adverse effects of treatment	9 (3, 0)
Immobility	7 (2, 3)
Others	23 (7, 6)
Total	219 (72, 6)

## Data Availability

The datasets generated and/or analyzed during the current study are not publicly available in order to preserve the anonymity of the respondents but are available from the corresponding author upon reasonable request.
